# Lack of Association between Measles Virus Vaccine and Autism with Enteropathy: A Case-Control Study

**DOI:** 10.1371/journal.pone.0003140

**Published:** 2008-09-04

**Authors:** Mady Hornig, Thomas Briese, Timothy Buie, Margaret L. Bauman, Gregory Lauwers, Ulrike Siemetzki, Kimberly Hummel, Paul A. Rota, William J. Bellini, John J. O'Leary, Orla Sheils, Errol Alden, Larry Pickering, W. Ian Lipkin

**Affiliations:** 1 Center for Infection and Immunity, Mailman School of Public Health, Columbia University, New York, New York, United States of America; 2 Division of Pediatric Gastroenterology and Nutrition, Massachusetts General Hospital, Boston, Massachusetts, United States of America; 3 Department of Neurology, Harvard Medical School and Departments of Neurology and Pediatrics and Learning and Developmental Disabilities Evaluation and Rehabilitation Services (LADDERS), Massachusetts General Hospital, Boston, Massachusetts, United States of America; 4 Department of Pathology of Harvard Medical School and Massachusetts General Hospital, Boston, Massachusetts, United States of America; 5 Measles, Mumps, Rubella, and Herpesvirus Laboratory Branch, Centers for Disease Control and Prevention, Atlanta, Georgia, United States of America; 6 Department of Histopathology, Trinity College Dublin, Dublin, Ireland; 7 American Academy of Pediatrics, Elk Grove Village, Illinois, United States of America; 8 National Center for Immunization and Respiratory Diseases, Centers for Disease Control and Prevention, Atlanta, Georgia, United States of America; National Institutes of Health, United States of America

## Abstract

**Background:**

The presence of measles virus (MV) RNA in bowel tissue from children with autism spectrum disorders (ASD) and gastrointestinal (GI) disturbances was reported in 1998. Subsequent investigations found no associations between MV exposure and ASD but did not test for the presence of MV RNA in bowel or focus on children with ASD and GI disturbances. Failure to replicate the original study design may contribute to continued public concern with respect to the safety of the measles, mumps, and rubella (MMR) vaccine.

**Methodology/Principal Findings:**

The objective of this case-control study was to determine whether children with GI disturbances and autism are more likely than children with GI disturbances alone to have MV RNA and/or inflammation in bowel tissues and if autism and/or GI episode onset relate temporally to receipt of MMR. The sample was an age-matched group of US children undergoing clinically-indicated ileocolonoscopy. Ileal and cecal tissues from 25 children with autism and GI disturbances and 13 children with GI disturbances alone (controls) were evaluated by real-time reverse transcription (RT)-PCR for presence of MV RNA in three laboratories blinded to diagnosis, including one wherein the original findings suggesting a link between MV and ASD were reported. The temporal order of onset of GI episodes and autism relative to timing of MMR administration was examined. We found no differences between case and control groups in the presence of MV RNA in ileum and cecum. Results were consistent across the three laboratory sites. GI symptom and autism onset were unrelated to MMR timing. Eighty-eight percent of ASD cases had behavioral regression.

**Conclusions/Significance:**

This study provides strong evidence against association of autism with persistent MV RNA in the GI tract or MMR exposure. Autism with GI disturbances is associated with elevated rates of regression in language or other skills and may represent an endophenotype distinct from other ASD.

## Introduction

Beginning in 1998, Wakefield and colleagues reported intestinal abnormalities, including reactive lymphoid hyperplasia in ileum, in children with autism and other developmental disturbances [1]–[8]. These findings, combined with parent-reported associations of timing of onset of behavioral abnormalities with MMR administration, led to the hypothesis that MMR contributed to autism pathogenesis [1]. Subsequent studies from this group reported MV RNA in bowel biopsies and peripheral blood mononuclear cells (PBMC) from children with ASD [9]–[12].

Over 20 epidemiologic studies reported no temporal relationship between MMR and ASD [13]–[33], and three studies found no MV RNA in PBMC of ASD children [34]–[36]; however, no published studies from other research groups have addressed whether MV RNA is present in bowel of ASD children with GI disturbances. Here we report independent, blinded analysis of ileal and cecal tissues from children with ASD and GI disturbances and children with GI disturbances but no neurological deficits for the presence of MV RNA in three laboratories, including the one where the original reports of an association between ASD and MV were obtained.

## Results

Forty-seven children were recruited. Six recruits did not complete the study: 3 potential cases dropped out prior to colonoscopy; 1 potential case and 2 potential controls completed colonoscopy but had incomplete clinical assessments. No differences were found in age, sex, or case-control status between study completers and non-completers. An additional 2 potential cases were excluded for failure to meet diagnostic inclusion criteria (below cutoffs for autistic disorder [AUT] on ADI-R); and 1 case was excluded because no bowel biopsy material was available. The final study population consisted of 25 cases (AUT/GI group) and 13 controls (GI control group) presenting consecutively for ileocolonoscopy who received at least one dose of MMR and completed all study procedures.

Age at biopsy was similar for cases and controls [median (interquartile range, IQR), cases, 5.5 (3.0) years; controls, 5.1 (3.0) years], as was the distribution of cases and controls across the three age strata. Girls were older at biopsy than boys (*P* = 0.01). There were no significant differences between cases and controls in their distribution by sex within the three age groups (*Table 1*).

**Table 1:** Subject characteristics.

SUBJECT CHARACTERISTIC		AUT/GI CASES	GI CONTROLS
**SEX**	Male	23 (92)	9 (69)
n (%)	*3–5 years*	*15*	*7*
	*6–7 years*	*6*	*1*
	*8–10 years*	*2*	*1*
	Female	2 (8)	4 (31)
	*3–5 years*	*0*	*1*
	*6–7 years*	*0*	*1*
	*8–10 years*	*2*	*2*
	All subjects	25 (100)	13 (100)
**ETHNICITY**	Caucasian	18 (72)	12 (92)
n (%)	Asian	4 (16)	0 (0)
	Hispanic	2 (8)	0 (0)
	African-American	1 (4)	1 (8)
**AGE STRATUM**	3–5 years	15 (60)	8 (61)
n (%)	6–7 years	6 (24)	2 (15)
	8–10 years	4 (16)	3 (23)
**AGE AT BIOPSY**	All subjects	5.5 (3.0)^a^	5.1 (3.0)
in years, median (IQR)			
**AGE AT FIRST MMR**	All subjects	15.3 (1.7)^b^	16.0 (4.9)
in months, median (IQR) [RANGE]		[12.2–22.8]	[5.6–20.5]
**TIME FROM LAST MMR TO BIOPSY**	All subjects	40.8 (26.7)^c^	39.8 (21.1)
in months, median (IQR) [RANGE]		[23.7–97.9]	[3.5–64.5]
**TOTAL NUMBER OF MMR VACCINES**	All subjects	20^d^	31
% receiving 2 doses			
**TOTAL NUMBER OF ALL VACCINES**	All subjects	17 (4)*	20 (1)
median (IQR) [RANGE]		[13–21]	[15–22]

a Mann-Whitney U, one-tailed, *P* = 0.67.

b Mann-Whitney U, one-tailed, *P* = 0.15.

c Mann-Whitney U, one-tailed, *P* = 0.50.

d *X^2^*, Fisher’s exact test, one-tailed, *P* = 0.36.

* Mann-Whitney U, one-tailed, *P* = 0.04.

The clinical indications for endoscopic/colonoscopic procedures commonly noted in both AUT/GI and GI groups included recurrent abdominal pain (RAP), gastroesophageal reflux, vomiting, and food allergies. Although the more subjective factor of RAP was frequently present in both cases (36%) and controls (38%), it was rarely the sole rationale for GI examination in either group (1 of 25 cases, or 4%; 2 of 13 controls, or 15%; *P* = 0.27).

Median age at receipt of first MMR was similar for cases [15.3 (1.7) months] and controls [16.0 (4.9) months]. The majority of study subjects were in the 3–5 year age stratum and below the age recommended for second MMR (4–6 years [37]); expectedly, 80% of cases and 69% of controls received only one MMR prior to the study (*P* = 0.36). Consistent with the older age of girls in the study, there was a trend toward a higher proportion of girls than boys receiving a second MMR (*P* = 0.13). None of the children received MV-containing vaccines other than MMR.

Clearance of MV depends on development of adaptive immunity. As cell-associated MV RNA may be present transiently after receiving MMR [38]–[39], timing of vaccination relative to biopsy was potentially important. Parental reports of timing of MMR receipt 6 months or more prior to biopsy were in accord with pediatric provider immunization charts for the final study population with the exception of one control boy whose immunization record revealed receipt of a second MMR 3.5 months prior to biopsy. This subject was retained in final analyses after determining results to be the same both with and without inclusion of his data. The median MMR-biopsy interval was similar for cases [40.8 (26.7) months] and controls [39.8 (21.1) months], and was not influenced by sex (*Table 1*). Older age at biopsy was associated with a longer MMR-biopsy interval, independent of case status (Spearman rank correlation, Rho = 0.65, *p*<0.0001).

Controls received a greater median number of all types of vaccines than cases [20 (1) vaccines vs. cases, 17 (4); *P* = 0.04, *Table 1*). Total number of vaccines received was not related to age or sex.

The study sample included two sibling pairs; three of these children were controls (2 males, 1 female) and one was a case (male). Data from sibling pairs were retained after determining that patterns of results were unaltered by sibling pair exclusion.

### Neuropsychiatric status

AUT diagnoses were confirmed for all cases. Absence of AUT, other ASD, or other developmental disturbances was confirmed for controls. For one control, ADI-R was incomplete; this subject was retained after determining that CDI and clinical assessment were consistent with typical development.

Median AUT onset age was 13.5 (7.0) months (*Table 2*). Cases had a high rate of CPEA-defined behavioral regression (loss of language and/or other skills following acquisition), 88%, compared to published rates of 20–40% for the general ASD population [27], [40].

**Table 2:** Onset of GI episodes and autism relative to MMR administration.

Timing of event	AUT/GI cases	GI controls
	(n = 25)	(n = 13)
First MMR vaccine	15.3 (1.7)^a^	16.0 (4.9)
*age in months*, *median (IQR)*		
First episode of GI disturbance	12.0 (17.5)^b^	2.0 (19.5)
*age in months*, *median (IQR)*		
MMR before GI onset *[n (%)]*	12 (48)^c^	3 (23)
MMR after GI onset *[n (%)]*	13 (52)	10 (77)
Autism onset	13.5 (7.0)	Not applicable
*age in months*, *median (IQR)*		
MMR before autism onset *[n(%)]*	13 (52)	Not applicable
MMR after autism onset *[n (%)]*	12 (48)	Not applicable
GI onset before autism *[n (%)]*	16 (64)	Not applicable
GI onset after autism *[n (%)]*	9 (36)	Not applicable
MMR before GI onset *[n (%)]*	5 (20)	Not applicable
<AND>		
GI onset before autism *[n (%)]*		

Key: *MMR*, Measles-Mumps-Rubella vaccine.

a Mann-Whitney U, one-tailed, *p* = 0.15.

b Mann-Whitney U, one-tailed, *p* = 0.29.

c *X^2^*, Fisher’s exact test, one-tailed, *p* = 0.13.

### Real-time RT-PCR assays

Prior to examination of study samples, performance of the four different primer sets (two for H gene, two for F gene) was evaluated for the 12 cloned target regions using synthetic RNA standards. A lower limit of detection of 50 RNA molecules per reaction was confirmed for each primer set in all laboratories.

All laboratories correctly identified all positive controls using pre-established criteria for positivity (positive results in at least two of three wells with at least one of the primer pairs for F and one of the primer pairs for H). All laboratories correctly identified all negative controls.

Concordance across laboratories was achieved in the initial round of real-time RT-PCR assays for all positive and negative results with the exception of a single study sample, an ileal biopsy from a control. An additional three samples, one ileal sample (from a control) and two cecal samples (one case, one control) yielded signal in at least one assay in one laboratory but did not meet criteria for positivity. All four samples were retested as below to resolve discrepancies.

As detailed above, only one sample met the pre-established definition of discordance; in this instance, an ileal sample from a control was positive with all four MV primer pairs in a single laboratory. Neither of the other two laboratories reported positive wells with any primer/probe combinations for this sample. The amplification product from this reaction was sequenced and determined to contain the engineered restriction site, confirming that it represented the synthetic transcript control. This sample was classified as negative. Aliquots of the three other samples that had yielded signal in one assay in a single laboratory were shipped to all three laboratory sites for retesting under new IDs. Two negative and one positive control were included to ensure blinding and monitor assay performance. Repeat testing of these three discordant samples with the F or H gene sequence primer/probe set responsible for the initial single positive finding failed to reproduce positive results in any of the three laboratories on the second round. In all three instances, results were negative on second round testing, including the one laboratory initially reporting positive results for a single primer pair.

### MV RNA in bowel biopsies

Analyses in all three laboratories found two ileal biopsy samples with MV F gene and H gene RNA: one from a boy in the AUT/GI group, the other from a boy in the control group. Real-time RT-PCR indicated a range of 2–7 molecules per PCR reaction, corresponding to approximately 50–500 MV RNA molecules per 100 ng of total RNA extract (*Table 3*). Sequence analysis confirmed that products of these samples were authentic. MV RNA was not detected in cecum of these subjects, or in ileum or cecum of any other subject. The presence of MV sequences was not associated with an AUT diagnosis (cases, 4%, controls, 8%).

**Table 3:** MV RNA detected by real-time RT-PCR.

ID	Study group	Sex	Age at biopsy (yr)	Age at MMR (mo)	Time since last MMR (mo)	GI region with MV sequences	Number of molecules per PCR reaction*			
							*mean*±*SD (laboratories positive for primer set)*			
							F gene		H gene	
							MeF	OLF	MeH	OLH
19	AUT/GI	M	4.71	15.9	40.6	Ileum	3.5±2.3 (Dub, CU, CDC)	1.7±0.7 (Dub, CU, CDC)	1.9±1.8 (CU, CDC)	NEGATIVE
35	GI control	M	3.98	20.5	27.2	Ileum	6.9±2.8 (Dub, CU, CDC)	3.3±1.6 (Dub, CU, CDC)	2.1±1.2 (Dub, CU, CDC)	2.1±0.7 (CU, CDC)

* Represents number of cDNA molecules per PCR reaction; each PCR reaction represents 100 ng total RNA from one sample.

Key:

*Dub*, Trinity College Dublin (Coombe Women's Hospital), Dublin.

*CU*, Columbia University, New York.

*CDC*, Centers for Disease Control and Prevention, Atlanta.

*MeF*, *MeH*, measles primer sets specific for F and H gene regions of MV, respectively (*Appendix S1*).

*OLF*, *OLH*, measles primer sets specific for F and H gene regions of MV, respectively [10] (*Appendix S1*).

Both subjects with positive samples had reactive lymphoid follicles (RLF). In the AUT/GI subject, RLF were present in both small and large intestine; the control had RLF restricted to colon. Endoscopy revealed inflammation in both subjects: the case had nonspecific gastritis; the control had acute distal esophagitis. Other cases and controls had RLF and/or inflammation in their upper and lower GI tracts, but MV sequences were not detected in their GI samples.

### Timing of MMR, GI episodes and AUT

If MMR is causally related to either GI disturbances or AUT it should precede their onset. Similarly, if GI disturbances contribute to AUT they should precede onset of AUT. We approached temporal relationships in the following manner: subjects with MMR administration and GI onset in the same month were considered to have MMR administration before the onset of GI episodes; subjects with GI episode and AUT onset within the same month were considered to have GI onset before AUT onset; and subjects with MMR and AUT onset within the same month were considered to have MMR onset before the onset of AUT.

There were no significant differences in the proportion of cases and controls with MMR before onset of GI episodes: 12 of 25 cases (48%) received MMR before GI episodes began as compared with 3 of 13 controls (23%; *P* = 0.13; *Table 2*). To examine whether the MMR-GI onset interval differed for cases and controls, survival analysis was pursued, using only those children with onset of GI episodes after MMR administration. Kaplan-Meier analysis showed no differences between cases and controls in latency from MMR to initial GI disturbances (Mantel-Cox logrank test).

To determine whether our data supported the hypothesis that GI pathology contributes to ASD pathogenesis, we examined the temporal relationship between MMR immunization, first GI episode, and AUT onset. If the putative relationship of MMR to GI pathology and AUT is valid, MMR must precede GI dysfunction and AUT, and GI dysfunction must precede AUT. If GI dysfunction contributes to AUT independent of MMR, it is necessary only that GI dysfunction precede development of AUT. *X^2^* analyses indicated no role for MMR in either the pathogenesis of AUT or GI dysfunction (*Table 4*). Only 5 of 25 subjects (20%) had received MMR before the onset of GI complaints and had also had onset of GI episodes before the onset of AUT (*P* = 0.03).

**Table 4:** Number and frequency of AUT/GI subjects receiving MMR before or after GI onset and with index GI episode before or after ASD.

ORDER OF EVENTS	CASES	
	(n = 25)	
	GI before ASD	GI after ASD
	*n (%)*	*n (%)*
MMR before GI	5 (31)†	7 (78)
MMR after GI	11 (69)	2 (22)

† *X^2^*, Fisher’s exact test, one-tailed, *p* = 0.03.

Cases first receiving MMR prior to onset of GI complaints were older at index GI episodes [21.0 (22.0) months] than cases receiving their first MMR after GI episodes already began [1.0 (12.0) months; p<0.0001; *Table 5*]. Conversely, cases with GI episodes preceding AUT onset had much earlier onset of GI problems than cases with initiation of GI episodes after onset of AUT [2.5 (13.0) vs. 30.0 (23.3) months, respectively; *P* = 0.001].

**Table 5:** Ages of AUT/GI subjects receiving MMR before or after GI onset and with index GI episode before or after ASD.

ORDER OF EVENTS	AGE AT EVENT			
	in months, median (IQR)			
	First MMR	First GI episode	ASD onset	GI biopsy
**MMR before GI** (n = 12)	15.1 (3.2)	21.0 (22.0)**	16.0 (5.5)	58.2 (32.1)
**MMR after GI** (n = 13)	15.8 (0.6)	1.0 (12.0)	12.0 (9.5)	74.8 (37.3)
**GI before ASD** (n = 16)	15.3 (1.0)	2.5 (13.0)*	13.5 (4.5)	64.5 (35.2)
**GI after ASD** (n = 9)	15.3 (4.3)	30.0 (23.3)	12.0 (13.0)	71.3 (34.8)
**MMR before GI**	15.0 (4.7)	15.0 (6.5)	16.0 (6.0)	52.2 (29.4)
<AND> **GI before ASD** (n = 5)				
***NOT*** ** MMR before GI**	15.3 (0.9)	12.0 (22.0)	12.0 (10.5)	68.4 (33.1)
<AND> **GI before ASD** (n = 20)				
**All AUT/GI cases** (n = 25)	15.3 (1.7)	12.0 (17.5)	13.5 (7.0)	65.6 (35.7)

** Mann-Whitney U, one-tailed, p<0.0001.

* Mann-Whitney U, one-tailed, p = 0.001.

## Discussion

We found no differences between AUT/GI and GI control groups in detection of MV sequences in RNA extracted from ileal or cecal biopsy specimens. Real-time RT-PCR assays with molecular controls engineered to allow differentiation of products arising from synthetic vs. *bone fide* MV RNA produced consistent results across three laboratories, with each laboratory site reporting less than 10 cDNA copies of MV F and H gene in ileal biopsies from one child with autism and one child without neurological disorder.

Our results differ with reports noting MV RNA in ileal biopsies of 75% of ASD vs. 6% of control children [10], [41]. Discrepancies are unlikely to represent differences in experimental technique because similar primer and probe sequences, cycling conditions and instruments were employed in this and earlier reports; furthermore, one of the three laboratories participating in this study performed the assays described in earlier reports. Other factors to consider include differences in patient age, sex, origin (Europe vs. North America), GI disease, recency of MMR vaccine administration at time of biopsy, and methods for confirming neuropsychiatric status in cases and controls. Participation in the current study required confirmation in cases of the presence of an AUT diagnosis and exclusion in controls of AUT or other developmental disturbances.

MV in MMR has been proposed to induce GI inflammation, increasing permeability to neuroactive chemicals that promote developmental neuropathology [42]–[43]. If this model is correct, MMR immunization should precede GI complaints, and both MMR and GI complaints should precede onset of ASD. We found the age at the time of exposure to MMR relative to onset of GI problems in cases and controls and the temporal order of MMR administration, GI episodes, and AUT onset in cases to be inconsistent with a causal role for MMR vaccine as a trigger or exacerbator of either GI disturbances or autism.

ASDs comprise a wide range of endophenotypes that may represent different routes to pathogenesis. The work reported here eliminates the remaining support for the hypothesis that ASD with GI complaints is related to MMR exposure. We found no relationship between the timing of MMR and the onset of either GI complaints or autism. We also could not confirm previous work linking the presence of MV RNA in GI tract to ASD with GI complaints. The origin, nature, and frequency of GI disturbances within the larger ASD population remain unclear; focused research strategies are required to define these endophenotypes and determine their significance for causal hypotheses.

## Materials and Methods

### Human subjects

Families of potential subjects were invited to participate if ileocolonoscopy with biopsy was specifically indicated as part of clinical care. Invited children were scheduled for upper and/or lower endoscopic procedures based on clinical imperative. Routine informed consent for clinical procedures was obtained by the gastroenterologist. Informed consent procedures detailed additional research procedures to be performed, and specific written permission was provided by consenting parents and guardians and children capable of providing assent (7 years or older). Study procedures were approved by the Institutional Review Boards of Partners/MGH, CU Medical Center, and the CDC and by the Ethics Committee of Coombe Women's Hospital prior to study initiation.

### Subjects

Children between 3 and 10 years of age were serially and prospectively recruited from the Pediatric Gastroenterology and Nutrition and LADDERS Clinics (MGH) in the years 2003 to 2005 into two groups if they had clinically significant GI disturbances requiring ileocolonoscopic examination and either: 1) presence of Autistic Disorder (AUT; suspected or assigned) (AUT/GI cases), or 2) absence of known or suspected developmental disturbances (GI controls). Potential controls were frequency-matched to potential AUT/GI cases within three age strata: 3–5, 6–7, or 8–10 years. Eligible children received at least one prior immunization containing MV vaccine strain. Children reported by parents to have received MV immunization within 6 months of planned biopsy were excluded.

### Clinical procedures

Neuropsychiatric status was established for all subjects by child neurologists, psychiatrists or developmental pediatricians (LADDERS) using Diagnostic and Statistical Manual-Fourth Edition, Text Revision (DSM-IV-TR [44]) diagnostic criteria. Cases failing to meet full DSM-IV-TR criteria for AUT (299.00 code) were excluded from further analysis, including subjects with diagnoses of any DSM-IV-TR pervasive developmental disorder (PDD) other than AUT (PDD-Not Otherwise Specified, Asperger's Disorder, Childhood Disintegrative Disorder, Rett's Disorder) or genetic syndromes associated with ASD features (Fragile X, tuberous sclerosis, neurofibromatosis, trisomy 21).

Data were obtained from parents by trained clinical raters using standardized data collection forms. Pediatrician records were acquired to confirm parent-reported dates, types, brands, and lot numbers of immunizations. All subjects were evaluated using the Autism Diagnostic Interview-Revised (ADI-R [45]) and Shortened CPEA Regression Interview, modeled on the MacArthur Communicative Development Inventory (CDI) (courtesy of Catherine Lord) [27], [46]–[49]. Certified raters administered the ADI-R to caregivers of cases to confirm DSM-IV-TR AUT diagnoses using established cutoffs [49]. Controls were evaluated in the same manner as cases to exclude subjects with ASD or other developmental disturbances.

Regression status (loss of language and/or other skills) was established according to well-validated CPEA algorithms [27], [48]–[49]. All diagnostic information (ADI-R, CDI, clinician diagnosis) was reviewed by a single pediatric neurologist to ensure consistency.

### Blinding

A randomized list of linked clinical and laboratory ID codes was prepared by the Biostatistics Core (CU) prior to study initiation and provided to MGH research assistants. The MGH clinical coordinator acquired, recorded and transmitted clinical data on case report forms identified only by clinical ID. To maintain the blind, patient samples were labeled at MGH with only the linked laboratory ID codes and shipped to the Laboratory Core (CU). Samples were maintained under laboratory codes for all assays at all three sites, including any repeat assays required to address inter-laboratory discordance. Linkage of clinical and laboratory ID codes remained with the Biostatistics group until inter-laboratory discordance was resolved and data quality control procedures were complete; a linked dataset was then prepared and final data analysis ensued.

### Sample acquisition

Biopsy material was obtained from terminal ileum and cecum under direct supervision of the team gastroenterologist. For analyses of MV RNA, four random samples were taken from superficial mucosae of ileum and cecum. Additional specimens were acquired at sites indicative of inflammatory GI lesions, if present. All samples intended for RNA analysis were frozen immediately in coded tubes in liquid nitrogen and stored at −70°C until shipment to CU on dry ice. Frozen biopsy specimens were stored in a dedicated −70°C freezer until RNA extraction to avoid inadvertent contamination. A portion of each clinical pathology sample also was retained under blind for histopathologic analysis.

### Preparation of RNA

Total RNA from bowel biopsies was obtained by acid guanidinium thiocyanate-phenol-chloroform extraction (TRI-Reagent, Molecular Research Center) at CU. Aliquots of RNA were prepared to ensure sufficient material for primary analysis at each of the three laboratory sites and for repeated analyses in the event results among sites were discordant. One 16 µg aliquot of RNA was created for each region (ileum, cecum, lesion) and sent to the three analytical laboratories for real-time RT-PCR detection of MV F and H gene sequences using two primer/probe sets each, as well as a control gene. To reduce the possibility that prior results might influence interpretation of repeat laboratory tests, additional aliquots of total RNA were coded with different laboratory IDs and stored at −70°C.

### Assay development

Three laboratory sites participated in MV RNA analyses: 1) Coombe Women's Hospital, Trinity College; 2) Center for Infection and Immunity, CU, New York; 3) Measles, Mumps, Rubella, and Herpesvirus Laboratory Branch, CDC, Atlanta.

Real-time RT-PCR assays employed four primer/probe sets: the sets originally described by Uhlmann *et al.* [10] for targeting F (fusion protein) and H (hemagglutinin protein) gene regions, and newly selected primer/probe sets targeting different regions in F and H gene sequences.

Synthetic MV transcripts were used as positive controls. These transcripts contained engineered mutations to allow distinction from *bona fide* MV sequences. A common cellular gene (β-actin) was used as a control for integrity of RNA template. Negative controls included reactions where water or normal human placental RNA was substituted for clinical sample RNA.

Additional details on primer pair design, positive and negative controls, and real-time RT-PCR assay calibration and procedures are provided in *Appendix S1*.

### Criteria for concordance

A positive finding in a single laboratory for each individual sample was defined as any result above the detection threshold (values above baseline and below a Ct of 45) in two of three triplicate reaction wells in a 96-well plate, for any single set of F or H primers, within that laboratory. The Biostatistics Core assessed coded data from each laboratory for positive results by GI region (ileum, cecum, lesion) and by primer pair, as well as for concordance of positive and negative findings across laboratories. All positive findings for a specific sample (RNA from one GI region, from a single subject) were first evaluated within each laboratory to determine whether both F gene and H gene MV sequences were represented (detection of RNA on the basis of two or more wells positive out of three, with either or both of the two primer/probe combinations for F and H gene regions). Concordance with respect to positive findings for a specific primer pair and GI region for an individual subject was next examined across laboratories; findings consistent in two or more laboratories for any primer pair/GI region were defined as concordant results. Samples for which at least two laboratories reported a positive finding with at least one F gene primer set and at least one H gene primer set were defined as positive for the presence of MV sequences (*Appendix S2*). Discordance across laboratories was defined as the presence of positive findings with at least one F gene primer set and at least one H gene primer set in a single laboratory.

### Data reporting and analysis

Assay results were reported under laboratory ID directly to the Biostatistics Core via a secure website. Prior to breaking the sample code, the Biostatistics Core reviewed consistency of results across the three sites for each individual sample. Concordance across laboratories was required for every positive and negative control as well as for each study sample. In instances where discordant results were noted across sites for any sample (including positive and negative controls), the Biostatistics Core notified the coordinating laboratory that additional aliquots of that sample were to be sent to each of the analytical laboratory sites for repeat assays. This process continued until any such inconsistencies were resolved. Products from positive reaction wells were sequenced to ensure the absence of engineered restriction sites and identify *bona fide* positives.

### Statistics

Group comparisons were conducted using nonparametric tests (Mann-Whitney U test; nominal α = 0.05) for continuous data deviating from normal distributions, and chi-square (*X^2^*) analyses for nominal data (Fisher's Exact Test to determine significance). One-tailed tests for significance were pursued unless otherwise indicated, given the study objectives of determining whether MV sequences were more likely to be found in biopsy tissues of cases than controls, as previously reported by Wakefield and colleagues and as consistent with the MMR hypothesis [1], [9]–[10]. Kaplan-Meier survival analysis was employed to examine whether the time between MMR exposure and onset of GI episodes differed for cases and controls, excluding subjects with index GI episodes occurring before MMR. A Mantel-Cox logrank test was used to compare survival curves after determining that assumptions of the test were met (e.g., independent, random samples; lack of correlation among covariates). Spearman rank correlation was used to evaluate whether older age at biopsy was associated with longer MMR biopsy intervals, independent of case status. StatView for Windows, version 5.0.1 (SAS Institute) and SPSS for Windows, version 15.0 (SPSS, Inc.) statistical software were employed for these analyses.

## Supporting Information

Appendix S1 Appendix S1 includes text, fig S1A, fig S1B, and table S1, with supplemental information regarding molecular methods and primer design
(0.19 MB DOC)

Appendix S2 Figure S2, study criteria and methods for resolving intra- and inter-laboratory disconcordance
(0.11 MB DOC)
