# Supplementary material for: Lack of Association between Measles Virus Vaccine and Autism with Enteropathy: A Case-Control Study
Source: PLoS One. 2008 Sep 4;3(9):e3140. doi: 10.1371/journal.pone.0003140 (PMC2526159; doi:10.1371/journal.pone.0003140)
Supplement: Appendix S1 — includes text, fig S1A, fig S1B, and table S1, with supplemental information regarding molecular methods and primer design (0.19 MB DOC) [file pone.0003140.s001.doc]

## Lack of association between measles virus vaccine and autism with enteropathy: a case-control study: Appendix S1

Mady Hornig^1§^, Thomas Briese^1^, Timothy Buie^2^, Margaret L. Bauman^3^, Gregory Lauwers^4^, Ulrike Siemetzki^1^, Kimberly Hummel^5^, Paul A. Rota^5^, William J. Bellini^5^, John J. O’Leary^6^, Orla Sheils^6^, Errol Alden^7^, Larry Pickering^8^, W. Ian Lipkin^1*^

August 13, 2008

**Contents Page**

**S1 Development of assays for MV RNA** 2

**S2 Positive and negative controls for real-time PCR 3**

**S3 Assay calibration and procedures 4**

**S4 Sequencing methods 4**

**References 4**

___________

^1^ Center for Infection and Immunity, Mailman School of Public Health, Columbia University, New York, NY, USA

^2^ Division of Pediatric Gastroenterology and Nutrition, Massachusetts General Hospital, Boston, MA, USA

^3^ Department of Neurology, Harvard Medical School and Departments of Neurology and Pediatrics and Learning and Developmental Disabilities Evaluation and Rehabilitation Services (LADDERS), Massachusetts General Hospital, Boston, MA, USA

^4^ Department of Pathology of Harvard Medical School and Massachusetts General Hospital, Boston, MA, USA

^5^ Measles, Mumps, Rubella, and Herpesvirus Laboratory Branch, Centers for Disease Control and Prevention, Atlanta, GA, USA

^6^ Department of Histopathology, Trinity College Dublin, Dublin, Ireland

^7^ American Academy of Pediatrics, Elk Grove Village, IL, USA

^8^ National Center for Immunization and Respiratory Diseases, Centers for Disease Control and Prevention, Atlanta, GA, USA

* Corresponding author (email, wil2001@columbia.edu; phone +1 212 342 9033; fax +1 212 342 9044)

^§^ Co-corresponding author (email, mady.hornig@columbia.edu; phone +1 212 342 9036; fax +1 212 342 9044)

**S1 Development of assays for MV RNA**

MeH and MeF primer pairs targeting the H and F gene of MeV, respectively, were designed using Primer Express Software (PE Applied Biosystems) based on the following GenBank sequence entries representing MV vaccine strains: AF266291, AF266290, AF266289, AF266288, AF266287, AF266286, AB046218, AB032167, and AB016162. Testing by real time RT-PCR demonstrated the capacity of the new primer/probe sets to detect vaccine strain and wild type sequences associated with outbreaks of human disease (data not shown). Alignment of primer/probe sets and wildtype sequences is shown in *Figure S1A and S1B (Real time RT-PCR primers for detection of MV F* and *H gene*, respectively*)*. Probes were labeled with the reporter FAM (6-carboxyfluorescein) and quencher TAMRA (6-carboxytetramethylrhodamine) (TIB MolBiol). Primer and probe sets used for detection of F and H gene transcripts and β-actin are listed in *Table S1*.


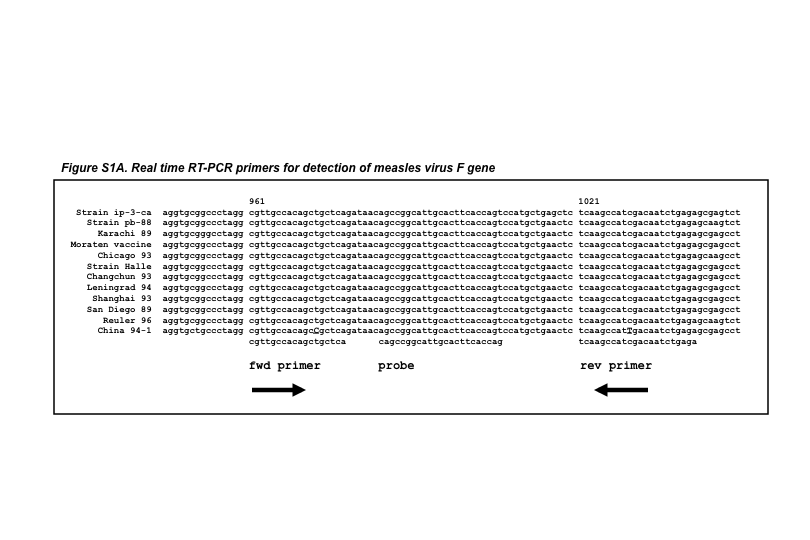


*
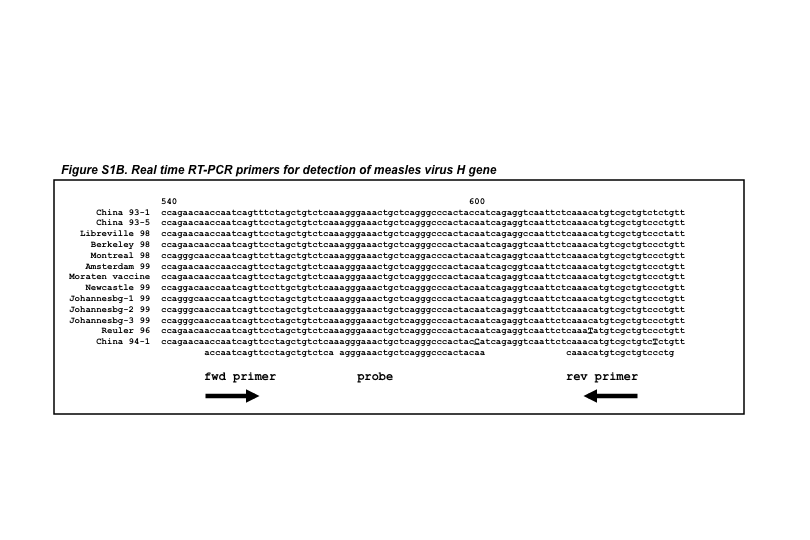
*


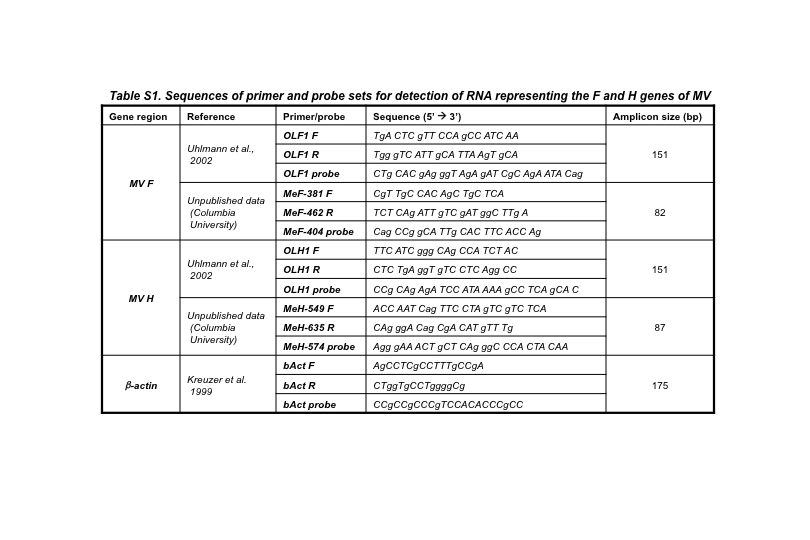


**S2 Positive and negative controls for real-time PCR**

To synthesize RNA standards for assay optimization, the plasmids MeF and MeH (pGEM-TEasy, Invitrogen) were linearized with Sal I. After phenol-chloroform/ethanol extraction, 750 ng of DNA were *in* vitro transcribed with T7 polymerase (mMESSAGE mMACHINE® T7 ULTRA Kit, Ambion). Linearized plasmid DNA and synthetic RNA transcripts were used in serial dilution with human placenta DNA (25 ng/μl, Sigma) or yeast tRNA (50 ng/μl, Sigma) as diluent to generate standard curves. Assay performance characteristics were validated using viral nucleic acid from three measles isolates: 1) vaccine strain virus (Moraten, a derivative of the Edmonston seed B strain; 2) MVi/Beijing.CHN/94-1 (Chinese wild type isolate); and 3) MVi/Reuler.LUX/17.96 (European wild type isolate). Standards were cloned for each of the respective gene target regions of these MV isolates.

Among these different standards, a detection threshold of at least 5 molecules plasmid standard per reaction was determined for the matching vaccine strain (Moraten); for the two divergent wild type isolates, sensitivity was reduced by 1-2 orders of magnitude, depending on the number of nucleotide mismatches in primers and probes (*Figure S1A* and *Figure S1B*). Using synthetic RNA transcripts generated from the Moraten vaccine strain sequence, a detection threshold of 50 molecules per RT-PCR reaction was determined.

**S3 Assay calibration and procedures**

All sites employed TaqMan Reverse Transcription Reagents (PE# N808-0234), TaqMan Universal PCR Master Mix (PE# 4304447) and either an ABI 7700 or ABI 7900 thermal cycler platform. Real-time PCR assay reagents (TaqMan Universal PCR Master Mix, primers, probes) and positive and negative quality control standards (DNA plasmid and RNA transcript standards; human placenta DNA, yeast tRNA, and water negative controls) were shipped to all three laboratories from CU prior to shipment of blinded RNA study samples. Quality control plates were run in each laboratory for calibration using synthetic cRNA standards prior to shipment of study samples. Results of calibration assays were transmitted directly to the Biostatistics Core and reviewed prior to shipment of coded study specimens to ensure integrity of password-protected data transfer procedures to a secure website and calibration of equipment and assay performance characteristics in each laboratory, including detection thresholds. RNA (2 μg total RNA/99 μl reaction) was subjected to reverse transcription (RT) with random hexamer priming. Five μl of cDNA from each RT reaction were run in triplicate in each of five real-time PCR assay plates (both primer/probe sets for F gene and H gene of MV, and the housekeeping gene), along with a full set of positive and negative controls on each plate. Thermocycling conditions for real-time PCR were: 2 min at 50°C, 10 min at 95°C (polymerase activation), 45 cycles of 15 sec each at 95°C (denaturation), and 1 min at 60°C (annealing, extension). Performance criteria were set to detection of 5-50 molecules and 50-500 molecules in the DNA and RNA dilution series, respectively.

**S4 Sequencing methods**

Amplification products were size-fractionated in 1.3% agarose gels and visualized by ethidium bromide staining at the end of the run. Products were sequenced after cloning into pGEM-Teasy plasmid vectors (Promega). Sequences were obtained for both strands by automated dideoxy-sequencing using BigDye Terminator Cycle Sequencing kits on ABI Prism Genetic Analyzer systems (Applied Biosytems).

**References**

1. Uhlmann V, Martin CM, Sheils O, Pilkington L, Silva I, *et al.* (2002) Potential viral pathogenic mechanism for new variant inflammatory bowel disease. Mol Pathol 55: 84-90.

2. Kreuzer KA, Lass U, Landt O, Nitsche A, Laser J, *et al*. (1999) Highly sensitive and specific fluorescence reverse transcription-PCR assay for the pseudogene-free detection of beta-actin transcripts as quantitative reference. Clin Chem 45: 297-300.
