# Supplementary material for: Lack of Association between Measles Virus Vaccine and Autism with Enteropathy: A Case-Control Study
Source: PLoS One. 2008 Sep 4;3(9):e3140. doi: 10.1371/journal.pone.0003140 (PMC2526159; doi:10.1371/journal.pone.0003140)

## Lack of association between measles virus vaccine and autism with enteropathy: a case-control study: Appendix S2

Mady Hornig^1§^, Thomas Briese^1^, Timothy Buie^2^, Margaret L. Bauman^3^, Gregory Lauwers^4^, Ulrike Siemetzki^1^, Kimberly Hummel^5^, Paul A. Rota^5^, William J. Bellini^5^, John J. O’Leary^6^, Orla Sheils^6^, Errol Alden^7^, Larry Pickering^8^, W. Ian Lipkin^1*^

August 13, 2008

**Contents Page**

**Figure S2** 2

___________

^1^ Center for Infection and Immunity, Mailman School of Public Health, Columbia University, New York, NY, USA

^2^ Division of Pediatric Gastroenterology and Nutrition, Massachusetts General Hospital, Boston, MA, USA

^3^ Department of Neurology, Harvard Medical School and Departments of Neurology and Pediatrics and Learning and Developmental Disabilities Evaluation and Rehabilitation Services (LADDERS), Massachusetts General Hospital, Boston, MA, USA

^4^ Department of Pathology of Harvard Medical School and Massachusetts General Hospital, Boston, MA, USA

^5^ Measles, Mumps, Rubella, and Herpesvirus Laboratory Branch, Centers for Disease Control and Prevention, Atlanta, GA, USA

^6^ Department of Histopathology, Trinity College Dublin, Dublin, Ireland

^7^ American Academy of Pediatrics, Elk Grove Village, IL, USA

^8^ National Center for Immunization and Respiratory Diseases, Centers for Disease Control and Prevention, Atlanta, GA, USA

* Corresponding author (email, wil2001@columbia.edu; phone +1 212 342 9033; fax +1 212 342 9044)

^§^ Co-corresponding author (email, mady.hornig@columbia.edu; phone +1 212 342 9036; fax +1 212 342 9044)


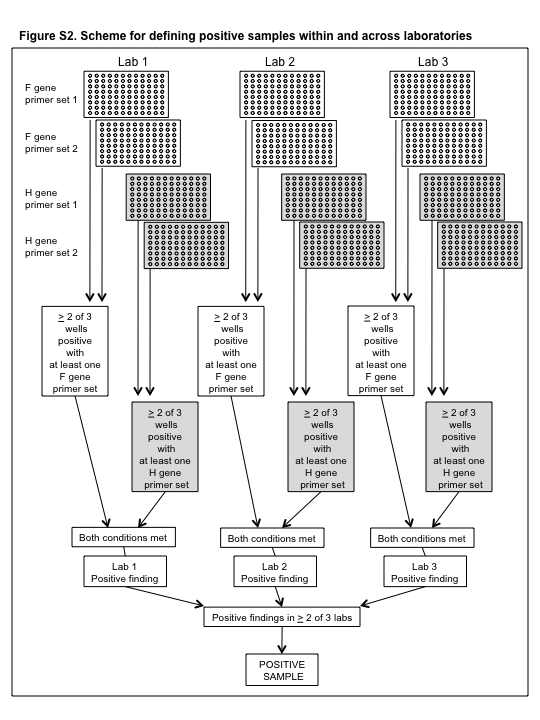

Supplement: Appendix S2 — Figure S2, study criteria and methods for resolving intra- and inter-laboratory disconcordance (0.11 MB DOC) [file pone.0003140.s002.doc]
